# Publisher Correction: Mid-infrared-perturbed molecular vibrational signatures in plasmonic nanocavities

**DOI:** 10.1038/s41377-022-00732-9

**Published:** 2022-02-24

**Authors:** Rohit Chikkaraddy, Angelos Xomalis, Lukas A. Jakob, Jeremy J. Baumberg

**Affiliations:** 1 NanoPhotonics Centre, Cavendish Laboratory, Department of Physics, JJ Thompson Avenue, University of Cambridge, Cambridge, CB3 0HE UK; 2 Present Address: Empa, Swiss Federal Laboratories for Materials Science and Technology, Laboratory for Mechanics of Materials and Nanostructures, Thun, Switzerland

**Keywords:** Nanophotonics and plasmonics, Photonic devices, Nanocavities, Terahertz optics, Single photons and quantum effects

Correction to: *Light: Science & Applications*

10.1038/s41377-022-00709-8 published online 19 January 2022

Following publication of this article^1^, it is reported that ‘Data availability’ section was missing. Hence the ‘Data availability’ is included in this Correction.

The original article has been updated.

**Data availability**

Source data can be found at 10.17863/CAM.79290.
