# Supplementary material for: Mid-infrared-perturbed molecular vibrational signatures in plasmonic nanocavities
Source: Light Sci Appl. 2022 Jan 19;11:19. doi: 10.1038/s41377-022-00709-8 (PMC8766566; doi:10.1038/s41377-022-00709-8)
Supplement: Supplementary file 1 — Supporting Information [file 41377_2022_709_MOESM1_ESM.pdf]

Supporting Information

Mid-infrared-perturbed Molecular Vibrational Signatures in Plasmonic Nanocavities

Rohit Chikkaraddy*[1], Angelos Xomalis [1] Lukas A. Jakob[1], and Jeremy J. Baumberg*[1]

[1] NanoPhotonics Centre, Cavendish Laboratory, Department of Physics, JJ Thompson Avenue, University of Cambridge, Cambridge, CB3 0HE, United Kingdom

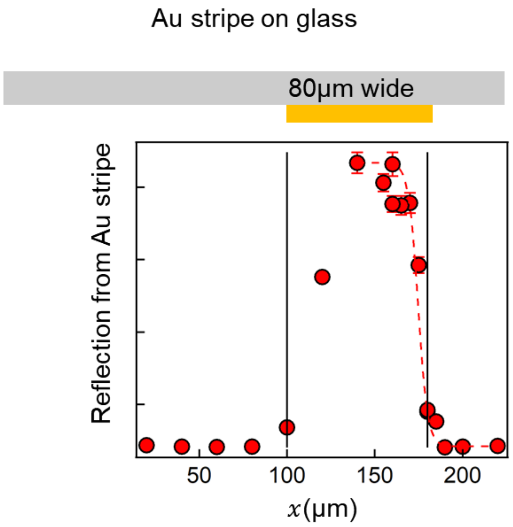

Fig. S1: Measurements of MIR laser spot size at λ = 10 µm. Laser spot is scanned across an 80 µm wide Au stripe fabricated on a glass cover slip placed on a computer-controlled stage. The reflection from the Au stripe is monitored and fit with a sigmoid function across the stripe edge to extract the MIR laser spot size focused by the Cassegrain objective.

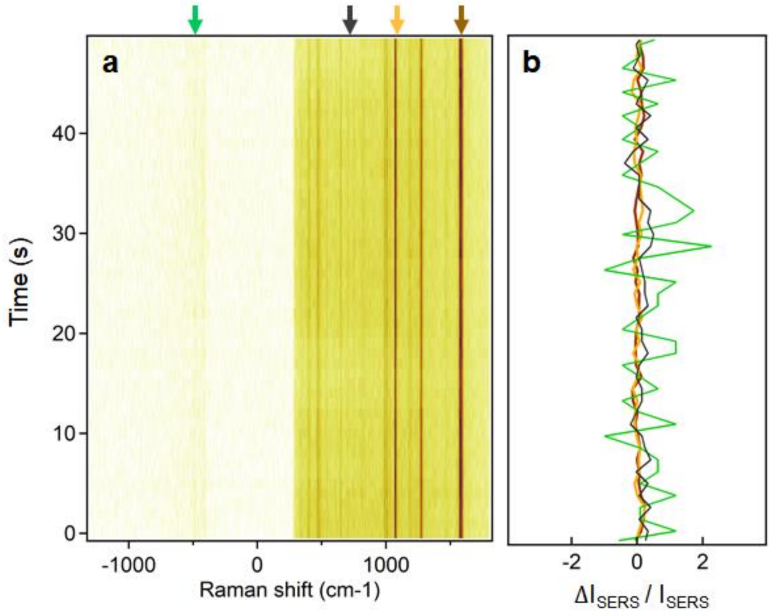

Fig. S2: (**a**) Time series SERS spectrum obtained from an individual NPoF in the absence of MIR light. (**b**) Extracted ΔI_{SERS} for BPT vibrational lines at: 1580 cm^{-1} (yellow), 1080 cm^{-1} (brown), Stokes ERS (grey) and anti-Stokes ERS (green), indicated by arrows in (**a**).

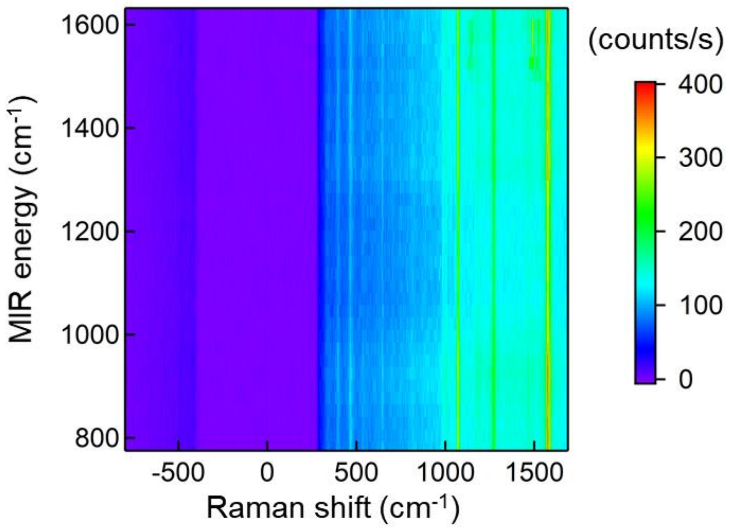

Fig. S3: SERS signal from an individual NPoF upon MIR frequency scanning (unprocessed spectra of Fig. 3a).

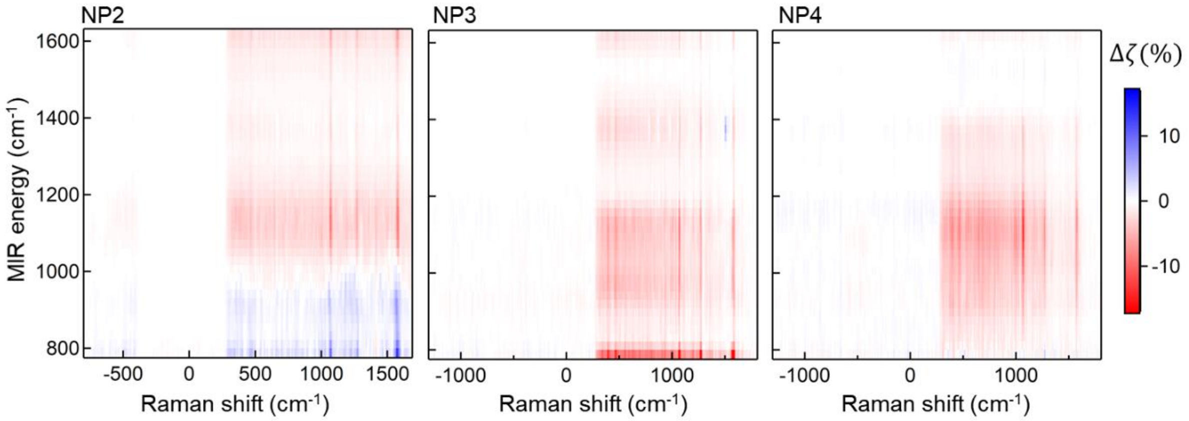

Fig. S4: Wavelength-dependent induced Δζ (%) in the SERS signal from several individual NPoFs when scanning the MIR frequency.

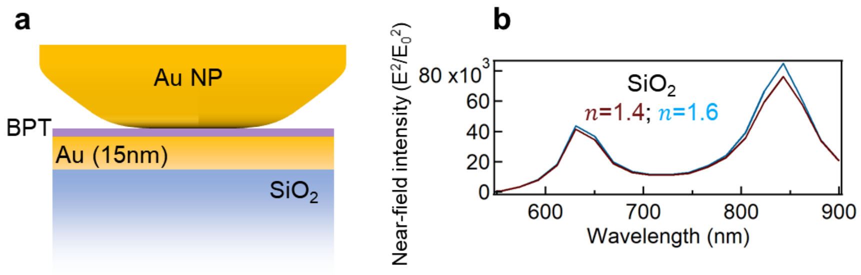

Fig. S5: (**a**) Schematic of simulated NPoF construct, explored for comparing different refractive indices (n) of SiO_2. (**b**) The near-field intensity at the gap centre as a function of wavelength. Note the very small change in near-field intensity for very large tuning of n, indicating that heating in SiO_2 cannot account for the observed signal shown in Fig. 2 of the main text.

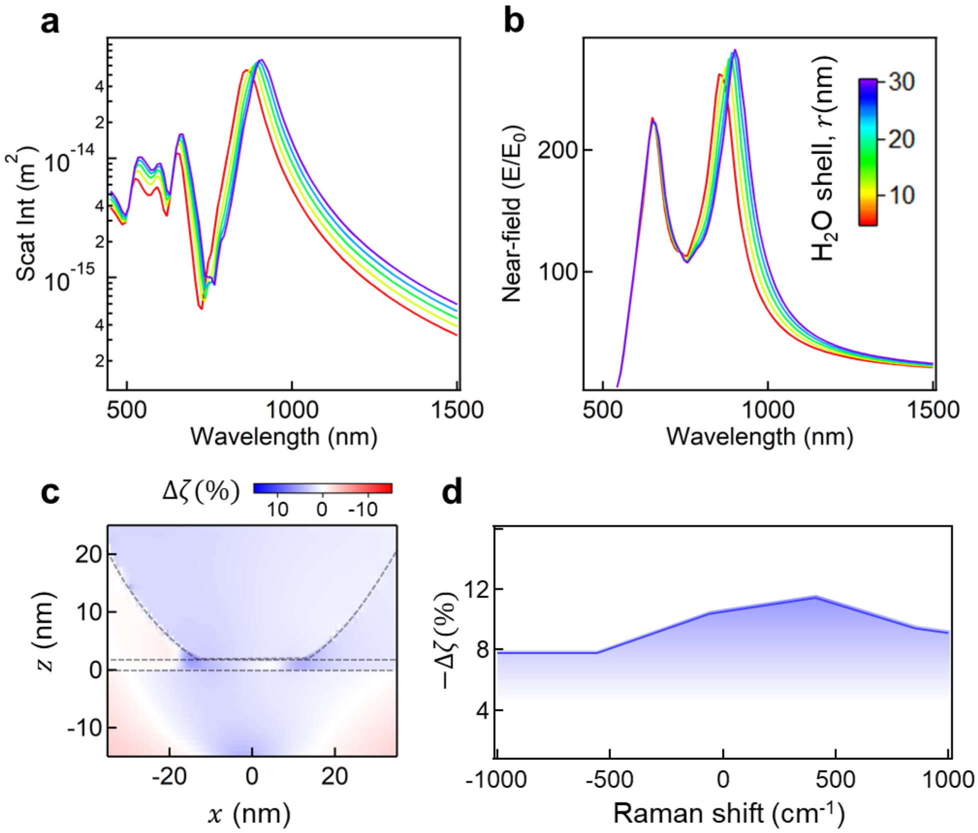

Fig. S6: Simulated (**a**) scattering and (**b**) near-field spectra of NPoF with varying thickness of water shell (r). (**c**) Calculated spatial map of $\Delta\zeta(\%)$ at 1080 cm^{-1} using the near-field intensity across the NPoF structure for r = 30 nm and high angle illumination from x-direction. (**d**) The calculated Raman $\Delta\zeta(\%)$ vs wavenumber, extracted from the optical field at the centre of the nanogap.

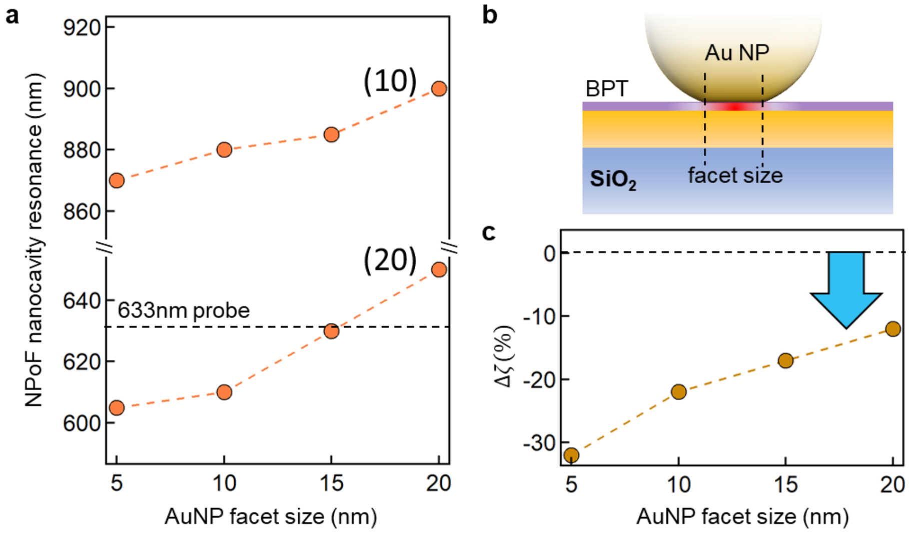

Fig. S7: Effect of NPoF resonance on the perturbed SERS. (**a**) Simulated (10) and (20) modes of the NPoF structure for (**b**) different facet sizes. Note that the (20) resonance peak moves through the probe wavelength at 633 nm. (**c**) The calculated SERS change $\Delta\zeta(\%)$, extracted from the optical field at the centre of the nanogap for different resonance positions. The sign of $\Delta\zeta(\%)$ is always negative irrespective of nanocavity resonance position.

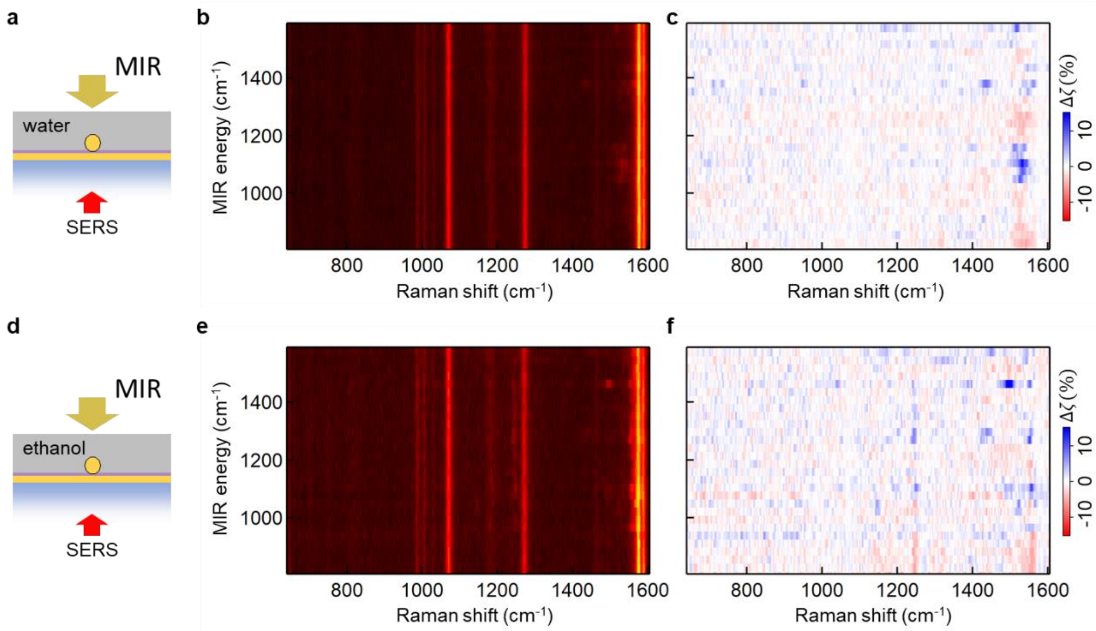

Fig. S8: Effect of different solvents on the perturbed SERS. (**a,d**) Schematic of NPoF samples immersed in (a-c) water and (d-f) ethanol solvents. (**b,e**) SERS signal from an individual NPoF upon scanning MIR frequency for each solvent. (**c,f**) Wavelength-dependent induced $\Delta\zeta$ (%) in the SERS signal from several individual NPoFs when scanning the MIR frequency.

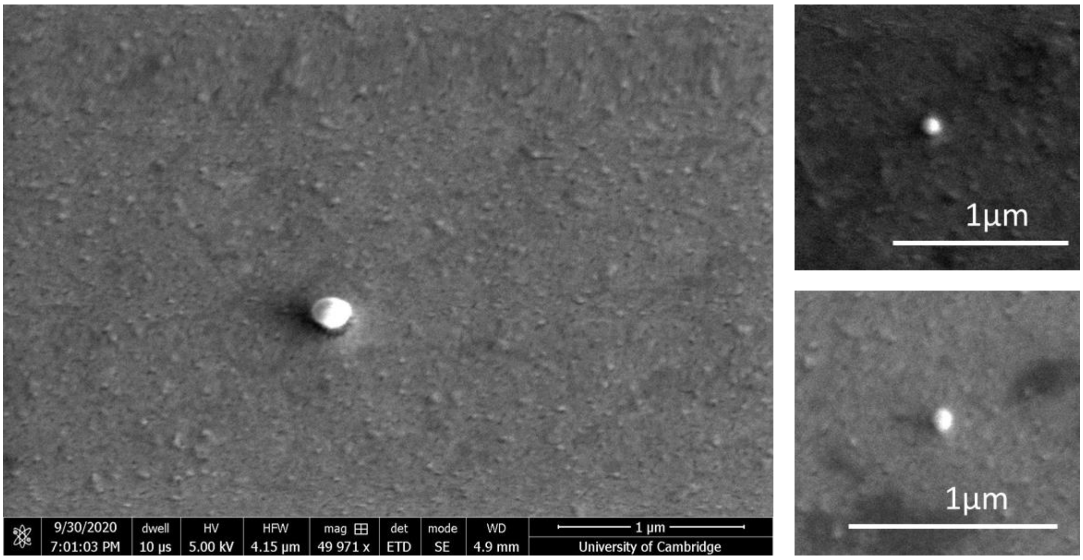

Fig. S9: Scanning electron microscope images of individual NPoF systems.

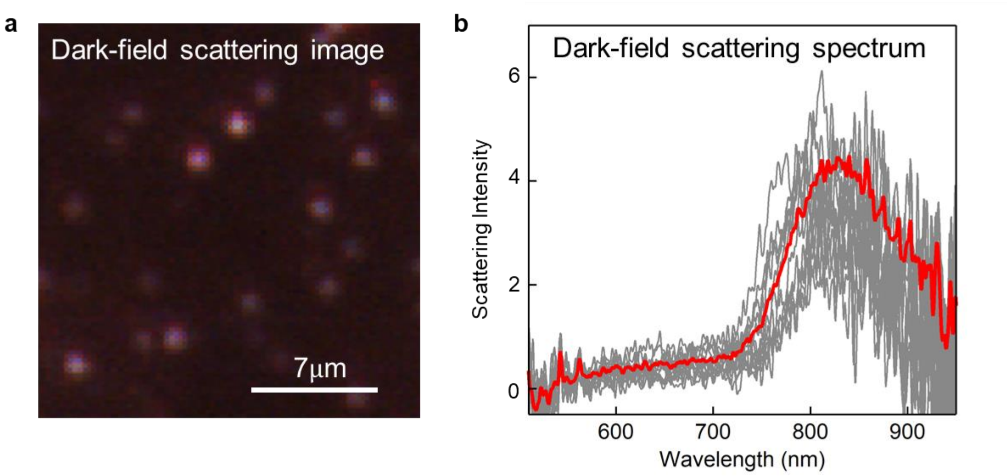

Fig. S10: Dark-field (**a**) image and (**b**) scattering spectra obtained from 10 different individual NPoF cavities. Red curve is the average spectrum, showing the (10) plasmon resonance at 820 nm.

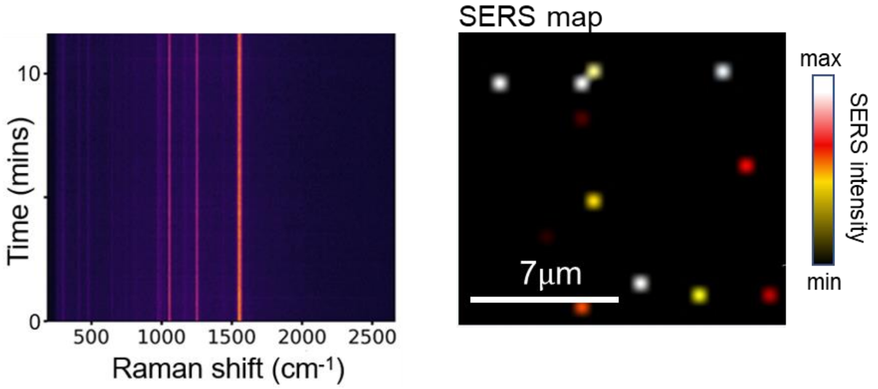

Fig. S11: (left) Repeated time-series of SERS spectra, showing the device stability. These are identical for different NPoFs, showing the consistent and uniform molecular layers formed. (right) Confocal SERS mapping of the NPoF cavities.

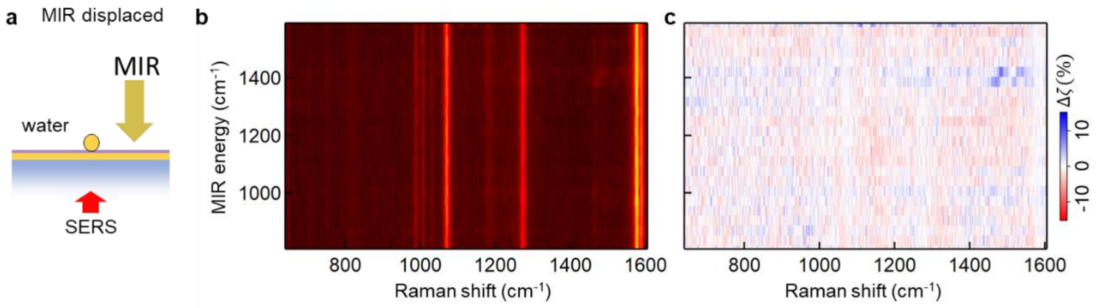

Fig. S12: (**a**) MIR focused away from the AuNP while visible laser beam remains focused on the AuNP to produce SERS. (**b**) Obtained SERS intensities are (**c**) not perturbed by the scanned MIR frequency.

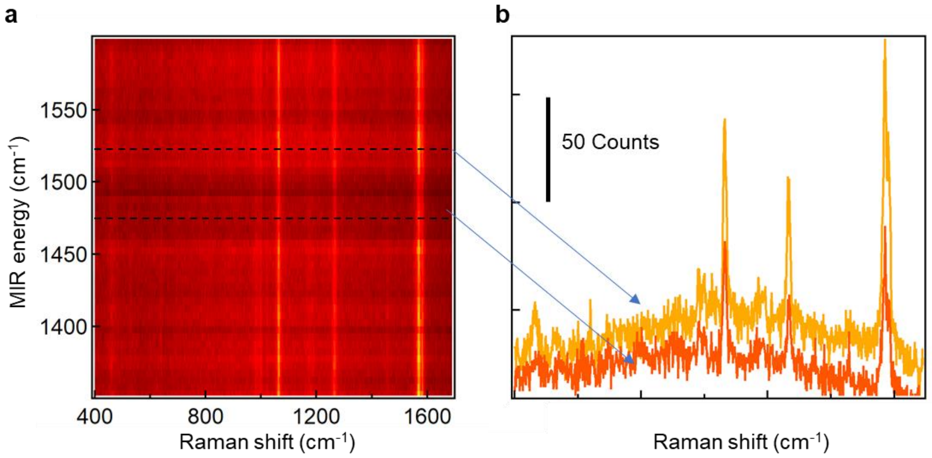

Fig. S13: (**a**) SERS from an individual polystyrene NPoF upon MIR frequency scanning (unprocessed spectra of Fig. 4b). (**b**) Extracted SERS spectra for MIR tuned to 1522 cm^{-1} and 1470 cm^{-1}.

Table S1: Different phenomena considered to explain observed modulation in SERS signal in the presence of MIR light.

**Phenomenon considered**	Why it does not fit experiments
**<u>Photothermal optical deflection</u>**<br>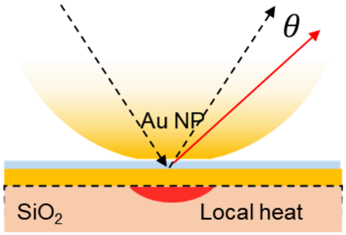<br><br>The time dynamics of MIR absorption-induced temperature rises in the system are estimated from $mC\frac{dT}{dt} = Q_a - Q_d$, where m and C represent the mass and specific heat capacity of the absorber, dT/dt is the change in temperature over time, and Q_a, Q_d are absorbed and dissipated heat energies. The heat absorption $Q_a = I_{MIR}\sigma$ and heat dissipation is governed by the gradient of heat $Q_d = hS[T(t) - T_0]$, where I_{MIR} is intensity of MIR light, σ is the absorption cross-section and h and S represent the heat transfer coefficient and effective transfer surface area from specimen to environment, respectively.<br><br>At equilibrium the sample maintains a constant temperature difference $[T(t) - T_0] = \Delta T$, $\frac{dT}{dt} = 0$ and $Q_a = Q_d$. This gives an estimated heat transfer parameter $hS = I_{MIR}\sigma/\Delta T$ from the full simulations (Lumericals, Heat module coupled with FDTD). For 20 µW µm^{-2} illumination of MIR light and 100 nm^2 of cross-section, we find hS = 4 nW K^{-1}.<br><br>The rate of change of temperature in the system is given by<br>$$[T(t) - T_0] = \frac{I_{MIR}\sigma}{hs}(1 - e^{-\frac{hs}{mC}t})$$<br>Considering the illumination volume (20 µm)^3 and C = 0.7 J (gK)$^{-1}$ (for SiO_2), the thermal decay rate $\left(\frac{mC}{hs}\right)$ is 0.3 s which is much slower than observed in our experiments. Even using instead the specific heat capacity of Au, C = 0.1 J (gK)$^{-1}$) will not give rates of a few 100 ns as observed in experiment.<br><br>Light scattering in NPoMs is determined by the nanocavity modes and SERS signals are out-scattered at high angles though (10) and (20) modes of the nanocavity. The change in temperature of AuNP do not affect these scattering angles.<br><br>Overall, the conventional photothermal signal mechanism is thus an unlikely explanation here.	- Rates of heat absorption and dissipation do not match with experiment.

<table>
<tr>
<td>

<u>Optical forces</u>

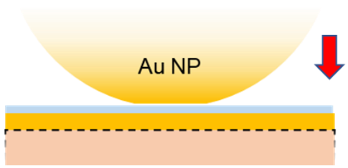

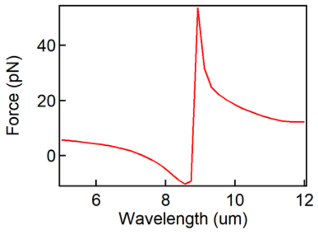

Optical forces in the dipole approximation are given by the polarizabilities of AuNP (α_{Au}) and substrate (α_{sub})

$$F_{\mathrm{opt}} \propto (\alpha_{\mathrm{Au}} \times \alpha_{\mathrm{sub}}) E_0^2 \,/ d^4$$

At 1 nm separation, with $\alpha = 4\pi\epsilon_0 R^3 \frac{(\epsilon-1)}{(\epsilon+2)}$ and using the complex ϵ for SiO_2 and Au, the wavelength dependent optical forces are dispersive line shapes.

</td>
<td>

- Wavelength-dependent line-shapes are dispersive, contrary to observed data.

</td>
</tr>
<tr>
<td>

<u>Thermal expansion</u>

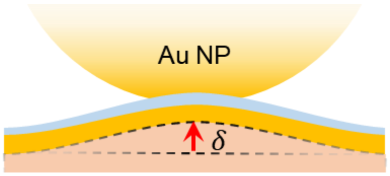

MIR light-induced thermal changes in the SiO_2, Au, and BPT are considered here.

Thermal expansion can change the visible-wavelength optics in two ways:

A. thermo-optic coefficient (τ)
B. linear expansion coefficient (η)

A. For SiO_2, τ = -1×10^{-4}°C^{-1}, which suggests that the change in refractive index (Δn) of the medium is <10^{-4} for a 1 K increase in temperature. The thermal simulations imply ΔT < 1 K (Fig. 6d). At the same time full-wave optical simulations show that the Δn needed for a 10% change in SERS signal is Δn > 0.2, a thousand times larger than this estimated Δn from τ.

B. For SiO_2, η = 80×10^{-6}°C^{-1}, which suggest that a 1°C increase in temperature results in a small expansion of SiO_2 perpendicular to the substrate. The amplitude of this undulation (δ), considering the calculated MIR heated spot size of 500 nm from the hot NP (Fig. 6d), is 0.04 nm. This is smaller than the radius of Au atom.

</td>
<td>

- Refractive index change needed is >0.2 which can only be achieved by laser powers >10 W.

- Undulations underneath the foil are too small, as the curvature needs to be on the scale of the AuNP radius to have any effect. Estimated local expansion of SiO_2 is much smaller than the size of a single Au atom.

</td>
</tr>
</table>
